# Increased resistance of gram-negative urinary pathogens after kidney transplantation

**DOI:** 10.1186/s12882-017-0580-z

**Published:** 2017-05-19

**Authors:** Johannes Korth, Julia Kukalla, Peter-Michael Rath, Sebastian Dolff, Marco Krull, Hana Guberina, Anja Bienholz, Benjamin Wilde, Stefan Becker, Birgit Ross, Olympia Evdoxia Anastasiou, Andreas Kribben, Oliver Witzke

**Affiliations:** 1 Department of Nephrology, University Hospital Essen, University Duisburg-Essen, Hufelandstrasse 55, 45147 Essen, Germany; 2 Department of Infectious Diseases, University Hospital Essen, University Duisburg-Essen, Hufelandstrasse 55, 45147 Essen, Germany; 3 Institute for Medical Microbiology, University Hospital Essen, University Duisburg-Essen, Hufelandstrasse 55, 45147 Essen, Germany; 4 Institute of Hygiene, University Hospital Essen, University Duisburg-Essen, Hufelandstrasse 55, 45147 Essen, Germany; 5 Department of Gastroenterology, University Hospital Essen, University Duisburg-Essen, Hufelandstrasse 55, 45147 Essen, Germany

**Keywords:** Urinary tract infection, Antimicrobial susceptibility, Klebsiella spp., E.Coli, kidney transplantation, Gram-negative urinary pathogens

## Abstract

**Background:**

Urinary tract infection is the most common complication after kidney transplantation. It can cause severe sepsis and transplant loss. Emergence of drug resistance among gram-negative urinary pathogens is the current challenge for urinary tract infection treatment after kidney transplantation.

**Methods:**

This study analyzes the antimicrobial susceptibility of gram-negative urinary pathogens after kidney transplantation from 2009 to 2012 at the Transplant Outpatient Clinic of the University Hospital Essen, Germany. Kidney transplant patients at the University Hospital Essen receive regular follow up examinations after transplantation. Midstream urines were examined for bacteriuria at each follow up visit.

**Results:**

From 2009 to 2012 15.741 urine samples were obtained from 859 patients. In 2985 (19%) samples bacterial growth was detected. The most frequently detected gram-negative bacteria were *E.coli* 1109 (37%), *Klebsiella spp*. 242 (8%) and *Pseudomonas aeruginosa* 136 (4.5%). *Klebsiella spp.* showed a significant increase of resistance to trimethoprim-sulfamethoxazole by 19% (*p* = 0.02), ciprofloxacin by 15% (*p* = 0.01) and ceftazidime by 17% (*p* = 0.004). *E.coli* and *P. aeruginosa* isolates presented no significant differences of antimicrobial susceptibility to the analyzed antibiotics.

**Conclusions:**

Antimicrobial resistance of *Klebsiella spp.* increased significant to trimethoprim-sulfamethoxazole, ciprofloxacin and ceftazidime from 2009 to 2012.

## Background

Urinary tract infection (UTI) is the most common infection after kidney transplantation [1–3]. It is also one of the most common causes for allograft failure, sepsis and mortality after transplantation. The risk for UTI after kidney transplantation is highest during the first year of transplantation with up to 60% [4]. Other risk factors are advanced age, female sex, diabetes mellitus and vesicoureteral reflux [5].

The incidence of drug resistant urinary pathogens seems to be increasing and has become a rising therapeutic challenge for clinicians. Drug resistant strains seem to have significant negative impact on the clinical course [6–9]. The rising incidence of resistant strains may be due to high antibiotic exposure. Antibiotic exposure could be identified as a risk factor for emerging resistance in gram-negative bacteria in other settings [10]. The antibiotic exposure after kidney transplantation is elevated due to treatment of asymptomatic bacteriuria (ASB) and pneumocystis pneumonia (PCP) prophylaxis.

The current treatment strategies of UTI after kidney transplantation are not defined or standardized. Most transplant center strategies include antibiotic treatment of ASB [11, 12]. The local UTI treatment strategy of the University Hospital Essen contains treatment of ASB as well [13].

This study investigates the prevalence of urinary pathogens and the resistance patterns in a cohort of kidney transplant patients during a period of 4 years (2009–2012) [14–16].

## Methods

### Study population and design

The present study was conducted at the University Hospital Essen, Germany. It compares retrospectively the antimicrobial susceptibility of gram-negative uropathogens isolated from kidney transplant recipients older than 18 years from 2009 to 2012. Patients with at least one sample per year with antibiotic resistance were assigned to the antibiotic resistant group. Analysis focused on the most common gram-negative pathogens that cause UTI including *E. coli, Klebsiella spp., and Pseudomonas aeruginosa* [17]. The evaluated antibiotics were chosen based on the use in the local guidelines for treatment of UTI and pneumocystis prophylaxis. *E. coli* and *Klebsiella spp*. were analyzed for susceptibility to ceftazidime, piperacillin/tazobactam, ciprofloxacin and trimethoprim-sulfamethoxazole. *P. aeruginosa* was analyzed for susceptibility to ceftazidime, piperacillin and ciprofloxacin. To answer if a shift of complicated UTI after renal transplantation occurred during observation period, the cases of urosepsis due to gram negative pathogens were documented and correlated to the number of urine cultures with gram negative pathogens (*E.coli*, *Klebsiella spp.*, *P. aeruginosa*). To determine recurrence of resistant urinary pathogens, the frequency of single and multiple detected *E.coli* and *Klebsiella spp.* isolates was recorded. The susceptibility to ceftazidime, piperacillin/tazobactam and ciprofloxacin was analyzed to evaluate if antimicrobial resistance was linked to recurrent pathogen detection. This study has been approved by ethics committee of the medical faculty of the University Duisburg-Essen (16–6952-BO).

### Study procedures

After transplantation all patients receive regular follow up examinations at the Transplant Outpatient Clinic of the University Hospital Essen. Midstream urines were obtained for urine culture at each follow up visit. Samples were transported to the Institute of Medical Microbiology within 4 h for analysis. Samples were cultured using standard media with semi quantification. Cultured bacteria were identified (MALDI-TOF) and susceptibility testing was performed using the VITEK system. Patients suffering UTI or ASB got empirical treatment according to the local UTI treatment strategy. All patients were treated with low-dose trimethoprim-sulfamethoxazole for pneumocystis (PCP) prophylaxis for at least 6 month after transplantation. To confirm the adherence to local guidelines the defined daily dose (ddd) from 2010 to 2012 of trimethoprim-sulfamethoxazole, ciprofloxacin, cefuroxime and amoxicillin/clavulanate was documented. For 2012 the ddd of nitrofurantoin was recorded as well. Data for nitrofurantoin from 2009 to 2011 are not available.

### Local UTI treatment strategy

*Enterococcus spp.* is frequently detected during the first 12 weeks after kidney transplantation [18]. Therefore empirical UTI treatment differs based on the time of occurrence after transplantation. Patients suffering from UTI symptoms during the first 2 months after transplantation received oral ciprofloxacin for 7 days. After the third month of transplantation, therapy was initiated with oral cefuroxime for 7 days. The antibiotic treatment was adjusted to the microbiological findings. Patients with urosepsis were initially treated with piperacillin/tazobactam and therapy was then adjusted according to the microbiological findings. The local guideline includes treatment of ASB according to results of the urinary culture test results [13].

### Definitions

Asymptomatic bacteriuria (ASB) was defined as pathogen detection >100.000 CFU/ml with presence of leukocytes (>100/μl) in urine in patients without UTI symptoms [13]. The diagnosis of UTI required the presence of symptoms like dysuria and pollakisuria combined with leucocyturia and/or positive nitrite test.

### Statistical analysis

The statistical analysis was performed with Graph Pad Prism V6.0. Chi-square test and fisher exact test were used between groups comparison.

## Results

From 2009 to 2012 a large number of 15.741 urine samples were obtained from 859 patients (324 (38%) male, 535 (62%) female). The average age of the patients was 54 years. Bacterial growth was detected in 2985 of 15.741 (19%) samples. In 2009 911/3931 (23%), in 2010 667/4310 (16%), in 2011 652/3566 (18%), and in 2012 755/3934 (19%) were tested positive.

### Species distribution of gram-negative uropathogens

The most frequently detected gram-negative bacteria were *E.coli* 1109/2985 (37%; 2009: *n* = 240; 2010 *n* = 300; 2011 *n* = 255; 2012 *n* = 314), *Klebsiella spp*. 242/2985 (8%; 2009: *n* = 68; 2010 *n* = 54; 2011 *n* = 42; 2012 *n* = 78) and *P. aeruginosa* 136/2985 (4.5%; 2009: *n* = 33; 2010 *n* = 32; 2011 *n* = 34; 2012 *n* = 37). No significant difference in the prevalence of the analyzed pathogens was detected (Table 1).

**Table 1:** Isolated gram negative urinary pathogens in kidney transplant patients from 2009 to 2012

Gram negative urinary pathogens in kidney transplant patients 2009–2012											
			*E. coli*	*Enterobacter cloacae*	*Citrobacter spp.*	*Klebsiella spp.*	*Morganella spp.*	*Proteus mirabilis*	*Serratia marescens*	*Pseudomonas aeroginosa*	*Stenotrophomonas maltophilia*
*n* = 544	2009	n/y	300	18	21	68	28	65	7	33	4
		%/y	55,1%	3,3%	3,9%	12,5%	5,1%	11,9%	1,3%	6,1%	0,7%
*n* = 444	2010	n/y	240	17	23	54	20	51	5	32	2
		%/y	54,1%	3,8%	5,2%	12,2%	4,5%	11,5%	1,1%	7,2%	0,5%
*n* = 424	2011	n/y	255	19	19	42	13	35	5	34	2
		%/y	60,1%	4,5%	4,5%	9,9%	3,1%	8,3%	1,2%	8,0%	0,5%
*n* = 538	2012	n/y	314	25	15	78	15	49	2	37	3
		%/y	58,4%	4,6%	2,8%	14,5%	2,8%	9,1%	0,4%	6,9%	0,6%
Increase/decrease			*ns*	*ns*	*ns*	*ns*	*ns*	*ns*	*ns*	*ns*	*ns*

*ns* not significant, *n/y* number of detected pathogens per year, *%/y* prevalence

### Defined daily doses (ddd) of antibiotics prescribed in 2010 and 2012

In total 7091 defined daily doses (ddd) of antibiotics were prescribed in 2010 and 7259 ddd were prescribed in 2012. The most frequently prescribed antibiotics were trimethoprim-sulfamethoxazole (2010 ddd: 3933, 55%; 2012 ddd: 3180, 44%), ciprofloxacin (2010 ddd: 857, 12%; 2012 ddd: 794, 11%) and cefuroxime (2010 ddd: 1347, 19%; 2012 ddd: 916, 13%). In 2012, nitrofurantoin was one of the most prescribed antibiotics with 13% (ddd: 924). Amoxicillin/clavulanate (2010 ddd: 81, 1%; 2012 ddd: 258, 4%) were prescribed less in both years. The remaining prescriptions (13 and 16%) of ddd in 2010 and 2012 were antibiotics without primary indication for UTI treatment. Overall, the total amount of prescribed antibiotics in 2010 was not different compared to the antibiotics prescribed in 2012. Trimethoprim-sulfamethoxazole and cefuroxim were less frequently prescribed in 2012 (−11 and −6%) as compared to 2010. The prescription of ciprofloxacin was not different between the years 2010 and 2012 (−1%). Amoxicillin/clavulanate prescriptions increased slightly fromm 2010 to 2012 with +3%. The amount of nitrofurantoin ddd in 2012 cannot be compared because data was not available in 2010.

### Antimicrobial susceptibility of *Klebsiella spp.*, *E.coli* and *Pseudomonas aeruginosa*

**Fig. 1:**
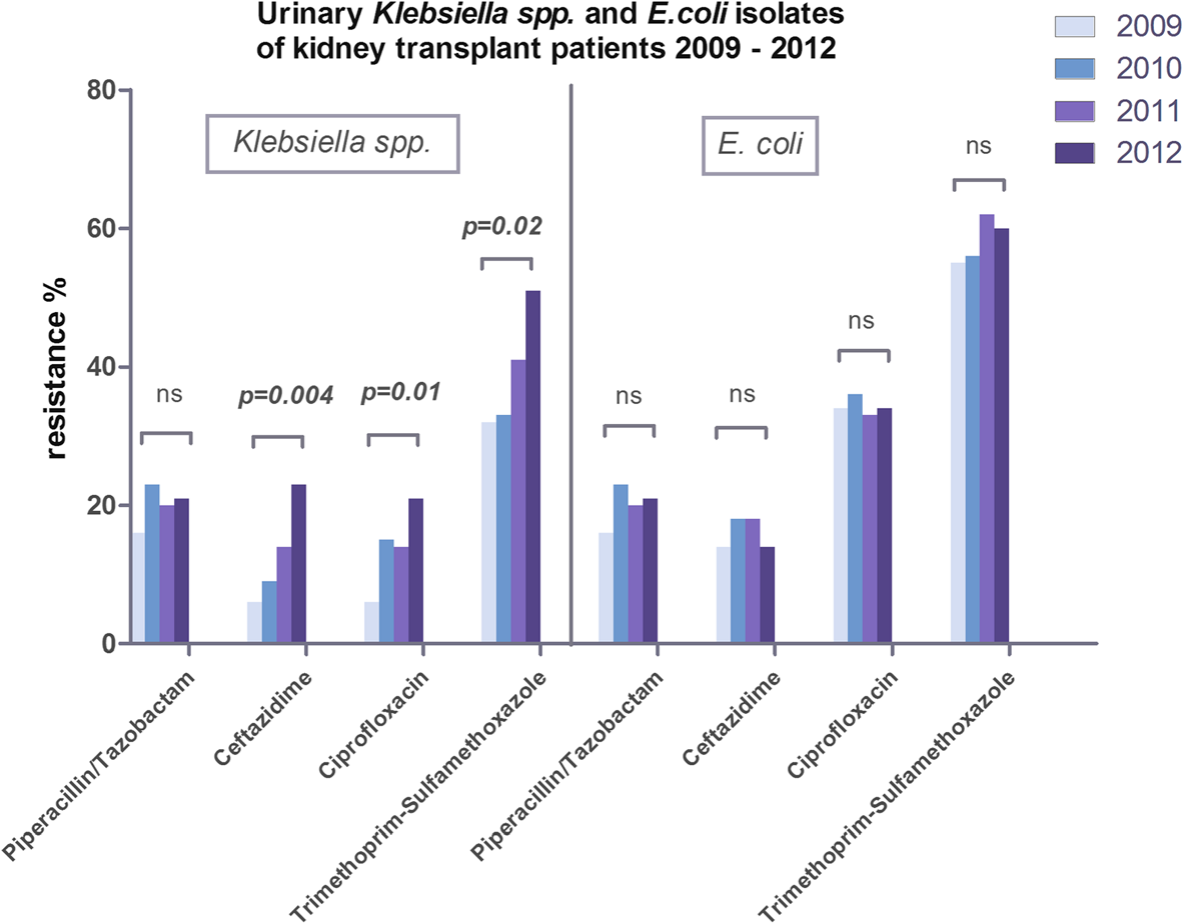
Displays the prevalence of urinary *Klebsiella spp.* and *E.coli* antibiotic resistance in kidney transplant recipients from 2009 to 2012. *Klebsiella spp.* resistance to ceftazidime, ciprofloxacin and trimethoprim-sulfamethoxazole increased significantly. The increase in resistance to piperacillin/tazobactam was not significant. *E.coli* isolates showed no significant increase or decrease in antibiotic resistance

*Klebsiella spp.* presented a significant increase of resistance to trimethoprim-sulfamethoxazole (*p* = 0.02; 2009 32%, 2010 33%, 2011 41%, 2012 51%), ciprofloxacin (*p* = 0.01; 2009 6%, 2010 15%, 2011 14%, 2012 21%) and ceftazidime (*p* = 0.004; 2009 6%, 2010 9%, 2011 14%, 2012 23%). The antimicrobial susceptibility to piperacillin/tazobactam was not significantly different (*p* = 0.50; 2009 16%, 2010 23%, 2011 20%, 2012 21%) (Fig. 1).

The *E.coli* isolates presented no significant difference of antimicrobial susceptibility to trimethoprim-sulfamethoxazole (*p* = 0.22; 2009 55%, 2010 56%, 2011 62%, 2012 60%), ciprofloxacin (*p* = 0.98; 2009 34%, 2010 36%, 2011 33%, 2012 34%), ceftazidime (*p* = 0.48; 2009 13%, 2010, 18%, 2011 18%, 2012 14%) and piperacillin/tazobactam (*p* = 0.11; 2009 16%, 2010 23%, 2011 20%, 2012 21%) (Fig. 1).

**Fig. 2:**
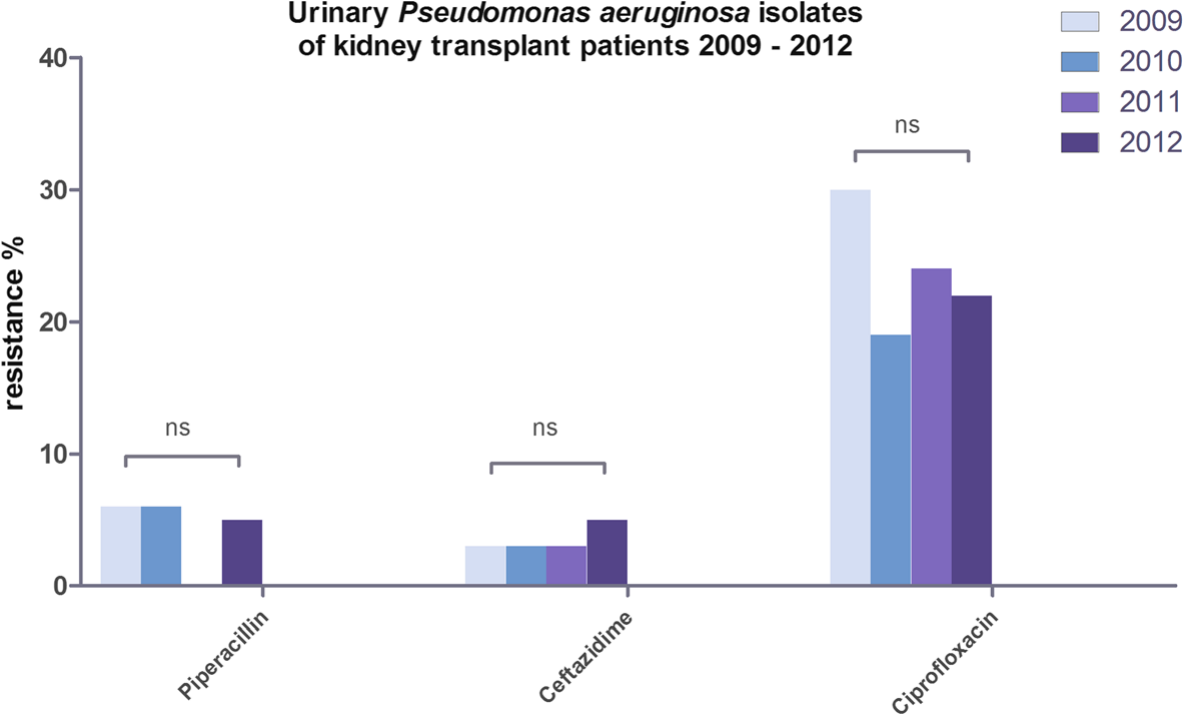
Displays the prevalence of urinary *Pseudomonas aeruginosa* antibiotic resistance in kidney transplant recipients from 2009 to 2012. No shift in resistance to piperacillin, ceftazidime and ciprofloxacin was observed

*P. aeruginosa* isolates presented no significant difference of antimicrobial susceptibility to ciprofloxacin (*p* = 0.40; 2009 30%, 2010 19%, 2011 24%, 2012 22%), ceftazidime (*p* = 0.62; between 3 and 5%) and piperacillin (*p* = 0.90; between 6 and 5%) (Fig. 2).

### ESBL phenotypes

**Fig. 3:**
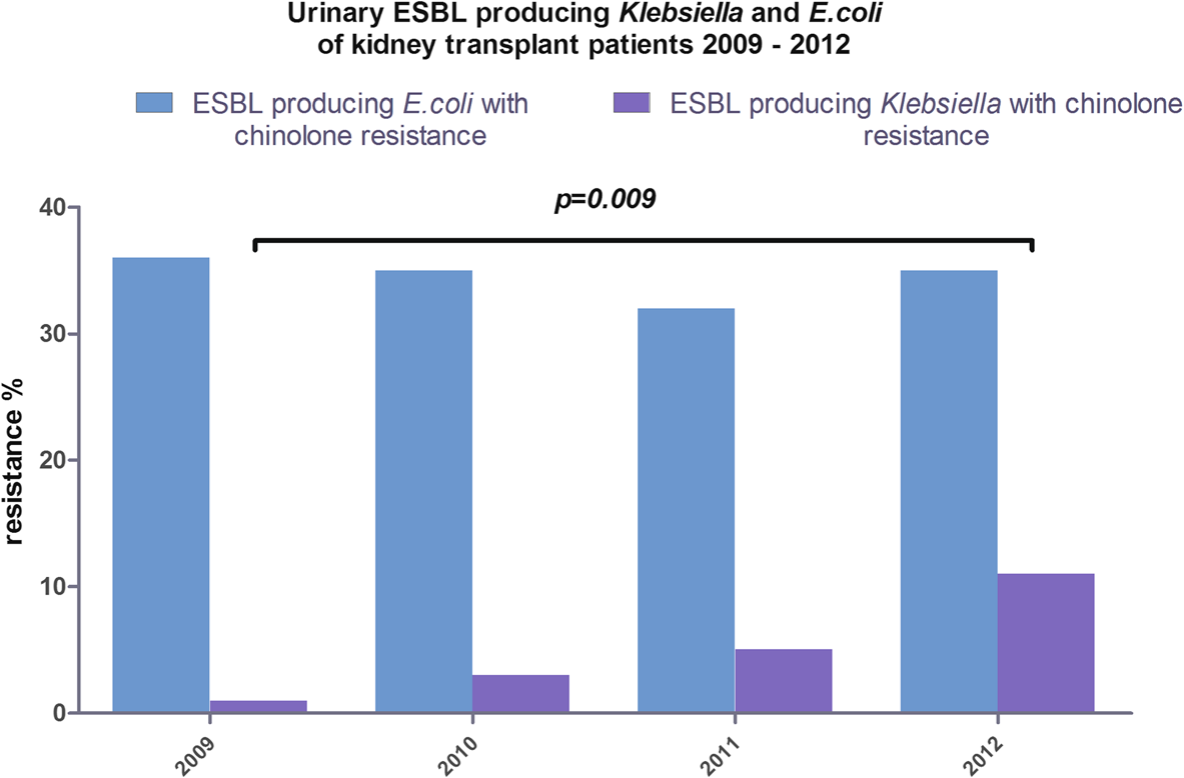
Displays the prevalence of urinary *Klebsiella spp.* and *E.coli* ESBL isolates in kidney transplant recipients from 2009 to 2012. The prevalence of *Klebsiella* ESBL isolates with chinolone resistance increased significantly. The prevalence of *E.coli* ESBL isolates remained constant

In 2009 44/3931 (1%) ESBL phenotypes were isolated. In 2012, the isolates with an ESBL phenotype remained stable with 48/3934 (1%). The number of ESBL producing *Klebsiella spp.* isolates and chinolone resistance increased significantly between 2009 and 2012 (*p* = 0.009; 2009 1/44 (2%), 2010 3/46 (7%), 2011 5/41 (12%), 2012 11/48 (11%) (Fig. 3). No significant difference was observed in ESBL producing *E.coli* with chinolone resistance (2009 36/44 (82%), 2010 35/46 (76%), 2011 32/41 (78%), 2012 35/48 (73%)). Between 2009 and 2012, only a single case with ESBL producing P*. aeruginosa* showing chinolone and carbapenem resistance was detected in our cohort of kidney transplant patients.

### Urosepsis after renal transplantation

In 51 renal transplant patients, urosepsis due to gram-negative pathogens (*E.coli*, *Klebsiella spp.*, *P. aeruginosa*) was diagnosed from 2009 to 2012. The number of cases with urosepsis normalized to the number of urinary cultures with gram-negative pathogens did not change between 2009 and 2012 (2009 2%; 2010 4%; 2011 4%, 2012 4%). In 36 out of 51 (70%) cases with urosepsis *E.coli* was detected, in 10/51 (20%) cases *Klebsiella spp.* was detected and in 5/51 (10%) cases *P. aeruginosa* was detected. The percentage of cases with *E.coli* detected in urosepsis showed an increase from 2009 to 2010 from 60 to 92%, and decreased to 2012 with 56%. Over the same time period, the percentage of patients with urosepsis and *Klebsiella spp.* increased from 10 to 38% (given as percentage of all cases with gram negative urosepsis in the respective year). In contrast, *P. aeruginosa* isolates in urosepsis cases decreased from 2009 with 30% to 2012 with 6%.

### Recurrent pathogen detection and antimicrobial susceptibility

*E.coli* was detected in 1109 urinary cultures during observation time from 2009 to 2012. In 654 cases, cultures were only once positive whereas 455 (41%) cultures were repeatedly positive in more than one urinary culture per year. Antimicrobial resistance to ceftazidime was detected in 45 (7%) of single positive cultures vs. 139 (31%) in multiple positive cultures. Antimicrobial resistance to ciprofloxacin was detected in 133 (21%) of all single positive cultures vs. 248 (54%) in multiple positive cultures. In addition, resistance to piperacillin/tazobactam was detected in 53 (8%) of all single positive cultures vs. 167 (36%) in multiple positive cultures. *Klebsiella spp.* were detected in 242 urinary cultures during observation time from 2009 to 2012. One hundred sixty-two (67%) were once positive and 80 (33%) were positive in multiple urinary cultures per year. Antimicrobial resistance to ceftazidime was detected in 9 (6%) of single positive cultures vs. 24 (30%) in multiple positive cultures. Antimicrobial resistance to ciprofloxacin was detected in 9 (6%) of single positive cultures vs. 25 (30%) in multiple positive cultures. Moreover, resistance to piperacillin/tazobactam was detected in 23 (13%) of single positive cultures vs. 25 (39%) in multiple positive cultures.

## Discussion

In the present study, we found that the species-specific prevalence of urinary tract infections remained stable between 2009 and 2012 in our cohort of renal transplant patients. Interestingly, the resistance pattern of *E.coli* and *P. aeruginosa* did not significantly shift over time. In contrast, the prevalence of ESBL producing and chinolone-resistant *Klebsiella spp.* increased steadily between 2009 and 2012.

In the literature *E.coli* is the most detected gram negative pathogen causing UTI with up to 38% [19, 20]. This can be confirmed with our present data. In addition no difference of the species distribution was detected from 2009 to 2012. The most frequent prescription of trimethoprim-sulfamethoxazole, ciprofloxacin and cefuroxime reflects the adherence to local guidelines for i. Pneumocystis pneumonia prophylaxis and ii. UTI. The lesser prescription of trimethoprim-sulfamethoxazole in 2012 compared to 2010 (−11%) is based on its primary use for Pneumocystis pneumonia prophylaxis in our center. It reflects the deceasing number of renal transplantations in Germany from 2010 to 2012 with −12,8% [21]. The high rate of nitrofurantoin ddd in 2012 shows the influence of clinical (practice) guidelines for uncomplicated UTI published in 2011 by the Infectious Diseases Society of America and the European Society for Microbiology and Infectious Diseases which recommends nitrofurantoin for uncomplicated UTI [22]. Furthermore nitrofurantoin may represent the use for ESBL-producing *E. coli,* as it shows to be an efficacious agent for UTI caused by ESBL-producing *E. coli* [23]*.*

Resistance of gram-negative strains increased during the last decades. Pathogens with distinct resistance against multiple groups of antibiotics are associated with increased mortality, longer hospital stays, and higher hospital costs [24, 25]. In our cohort, the prevalence of multi-resistant *Klebsiella spp*. increased steadily whereas *E.coli* as well as *P. aeruginosa* showed no significant change in resistance pattern. The increase of multi-resistant *Klebsiella spp*. isolates did not seem to have an impact on the number of urosepsis cases per year in our cohort, although the amount of *Klebsiella spp.* isolated in urosepsis cases increased steadily. Interestingly, increasing resistance of gram negative isolates has been observed in other cohorts, too [6, 16, 26–28]. In general, previous antibiotic exposure and increased broad-spectrum antibiotic consumption could be identified as risk factors for increasing resistance. After solid organ transplantation (SOT) additional risk factors are delayed graft function, diabetes mellitus and relapsing UTI [26, 29, 30]. Therefore we hypothesize that rising antimicrobial resistance of *gram negative pathogens* could be influenced by the present UTI treatment strategy.

In contrast to other reports, the shift of resistance pattern was limited to *Klebsiella spp*. in our cohort; the resistance pattern of other gram-negative pathogens remained unchanged over time. The reason why increased antibiotic resistance was limited to *Klebsiella spp.* isolates in our cohort remains unknown.

In our cohort, recurrent pathogen detection was linked to a higher rate of antimicrobial resistant *E.coli* and *Klebsiella spp.* detection. Recurrent detection of resistant pathogens may be due to insufficient first-line treatment, since quinolones and cephalosporins are included in the local UTI treatment guideline. Further more in literature MDR pathogens are frequent in kidney recipients with recurrent UTI [19]. This could be another explanation for the association of resistant pathogen detection in multiple urinary cultures, because renal transplant patients with recurrent or persistent UTI receive intensive follow up monitoring including repeated urinary cultures.

To challenge pathogen resistance, the reduction of antibiotic exposure is currently under discussion. The impact of the rational use of antibiotics on antimicrobial susceptibility remains controversial. A swedish study could not show an influence of rational antibiotic use on penicillin-resistant *Streptococcus pneumoniae,* although reduction of antibiotic use could be observed [31]. One prospective study of kidney transplant recipients with ASB showed no greater incidence of multi-drug resistant pathogens in patients receiving treatment for ASB compared to patients remaining untreated [32]. However, this study was limited to 115 patients and a follow up of 24 months.

Though, the majority of studies showed that resistance can be reduced by introducing antimicrobial stewardship programs [33, 34]. The stewardship programs recommend the use of the antibiotics with the narrowest spectrum possible. In addition limitation of the therapy duration is recommended.

However, in patients after SOT, reduction of antibiotic exposure is challenging. Trimethoprim-sulfamethoxazole prophylaxis is used for Pneumocystis pneumonia prophylaxis. The prophylaxis is crucial for survival after SOT [35]. Therefore it cannot be suspended, although it is associated with increased amoxicillin and trimethoprim-sulfamethoxazole resistance in gram-negative bacteria [36]. Even in our study, resistance of *Klebsiella spp.* isolates to trimethoprim-sulfamethoxazole increased significantly.

There are additional strategies to reduce antibiotic exposure and to strengthen rational antibiotic treatment. The differentiation between UTI and ASB should be implemented in future strategies. UTI after renal transplantation has a great impact on the transplant outcome. Gołębiewska et al. evaluated the influence of UTIs on renal graft function in a cohort of 209 adult renal transplant recipients. They observed 322 UTI episodes in 111 patients, including asymptomatic bacteriuria, lower and upper UTIs and urosepsis. Graft function was significantly worse in patients suffering from UTI [37]. Furthermore Pelle et al. investigated in a cohort of 177 renal transplant recipients that patients with upper UTIs (acute pyelonephritis) exhibited a decrease in creatinine clearance compared to patients with uncomplicated UTI 1 year after transplantation, which was still persistent 4 years after transplantation [18]. In addition Dharnidharka et al. investigated that the risk for graft loss after early UTI was elevated in a cohort of under aged renal transplant patients [38]. Therefore treatment for UTI is warrantable. In the context of “choosing wisely”, the antibiotic treatment of ASB is controversial [39]. A Cochrane Renal Group’s Specialized Register analysis could not find a clinical benefit of ASB treatment [40]. Even in the cohort of kidney transplant patients, treatment strategies for ASB are currently changing. However, untreated ASB within the first 3 months of transplantation has been identified as an independent risk factor for acute cellular rejection. [41]. In contrast ASB is not associated with short-term graft function impairment [42]. Therefore, treatment should be considered early after transplantation but maybe obsolete at a later time point [43]. Alternatively, patients with ASB could be monitored. Treatment should be initiated in case of decreasing kidney function or UTI symptoms [41]. This strategy may prevent unnecessary antibiotic exposure.

## Conclusions

This study found a concerning increase of antimicrobial resistance to commonly used antibiotic agents among gram-negative uropathogens isolated from kidney transplant recipients during an observation period of 4 years (2009–2012). Antimicrobial resistance of *Klebsiella spp.* to trimethoprim-sulfamethoxazole, ciprofloxacin and ceftazidime increased significantly from 2009 to 2012. Local treatment strategies of urinary tract infections and PCP prophylaxis may have influenced increased antimicrobial resistance.

## Abbreviations

ASB: asymptomatic bacteriuria
CFU: colony forming units
ddd: defined daily dose
ESBL: Extended Spectrum β Lactamase
PCP: *Pneumocystis* pneumonia
SOT: solid organ transplantation
spp.: species
UTI: Urinary tract infection
